# To Explore the Utility of Leaf Morphological, Color, and Chlorophyll Traits in Assessing Inter-Cultivar Variations Among Six Tea Plant Cultivars

**DOI:** 10.3390/biology15151283

**Published:** 2026-08-04

**Authors:** Pengliang Pan, Shibao Guo, Ruifei Wang, Zhou Zhou, Hongmin Liu, Hongzhong Shi, Gailing Zhang

**Affiliations:** 1 College of Agronomy, Xinyang Agriculture and Forestry University, Xinyang 464000, China; panpl@xyafu.edu.cn (P.P.); ruifeiwang@126.com (R.W.); 2021180005@xyafu.edu.cn (Z.Z.); 1998180043@xyafu.edu.cn (H.L.); 1988180008@xyafu.edu.cn (H.S.); zhangglxyafu@163.com (G.Z.); 2 Dabie Mountain Laboratory, Xinyang 464000, China

**Keywords:** *Camellia sinensis*, cultivar identification, leaf morphology, chlorophyll content, discriminant analysis, digital image analysis, genetic similarity

## Abstract

Reliable traits are needed for identification of tea (*Camellia sinensis*) cultivars, yet the stability of leaf morphology and color across leaf positions remains unclear. This study evaluated inter-cultivar variation and positional stability in leaf morphological, RGB color, and SPAD traits in six predominant cultivars. One-year-old shoots were sampled in a completely randomized design, and five fully expanded leaves below the apical bud were analyzed. SPAD values were measured with a chlorophyll meter, and scanned images were used to extract contour and RGB traits. Data were analyzed using ANOVA, correlation analysis, PCA, and discriminant analysis. Leaf morphology differed among cultivars and leaf positions, with significant cultivar-by-position interactions; however, the width-to-length ratio differed among cultivars but remained stable across positions in these cultivars. SPAD values increased with leaf position and were strongly associated with RGB components, being negatively correlated with R and G and positively correlated with B. Morphological traits explained 52.988% of total variance in PCA and yielded 64.6% overall classification accuracy, with LaoHan showing the highest accuracy (83.3%). Misclassification was concentrated among genetically similar cultivars. These findings suggest that stable leaf shape proportions and SPAD–RGB relationships provide useful descriptors, whereas genetic relatedness limits morphology-based cultivar identification under the present conditions.

## Simple Summary

In order to clarify the role of morphological and color characteristics of tea leaves in tea cultivar identification, this study analyzed the differences among six main local tea cultivars by acquiring the morphological, color, and chlorophyll characteristics of leaves at different positions on one-year-old tea branches. The results showed that there were significant differences in leaf morphology between different cultivars and leaf positions, and there was a significant interaction between cultivar and leaf position. The leaf width-to-length ratio showed significant differences among different cultivars, but remained relatively stable across various leaf positions in this study. Chlorophyll content increased with the increase in leaf position, and showed a significant correlation with color characteristics. The morphological characteristics of leaves explained more than half of the variation, and the highest correct discrimination rate for tea cultivars could reach 83.3%. Therefore, the above parameters have important reference values for identification of tea cultivars with different genetic backgrounds.

## 1. Introduction

Tea tree (*Camellia sinensis*) has a long cultivation history in China, where numerous varieties have been developed and disseminated through prolonged agricultural practice. In the Xinyang tea-growing region of Henan Province, repeated historical introductions and long-term cultivation have resulted in a mixed planting system composed of multiple varieties. Over extended periods of natural growth, some varieties have exhibited morphological variations or genetic instability [1,2], leading to a highly complex genetic background across large tea-growing areas. Within the context of precision agriculture, improving the efficiency of tea cultivar classification, tea garden monitoring, and automated tea harvesting remains a substantial challenge [3]. Accurate identification of tea plant varieties is therefore a prerequisite for improving automation efficiency.

According to previous research studies, molecular approaches based on plant genetic material have become one of the most accurate methods for discriminating plant varieties and cultivars [4,5]. With advances in plant phenotyping technologies, spectral traits have also emerged as important indicators for tea cultivar identification in recent years [6]. When combined with deep learning, these methods have made field identification of tea cultivars feasible [7]. However, the acquisition and analysis of molecular and spectral data are often time-consuming and costly.

With the development of image processing and analysis technologies, external morphological traits and visible-light RGB images acquired using conventional optical devices have played an important role in animal and plant classification [8] and in pest and disease diagnosis [9,10,11]. These approaches are widely regarded as low cost, efficient, and practical.

Plant chlorophyll content generally reflects physiological status and responses to abiotic stress [12]. Quantified by SPAD values, it has been widely used in plant health assessment, early warning of plant diseases, and breeding of disease-resistant varieties [13,14,15,16]. Meanwhile, image-based techniques that extract RGB values from plant leaves not only support crop yield estimation [17], but also facilitate monitoring of the dynamics of different plant, such as farmland weeds [18]. When RGB values are correlated with SPAD values, they can be further applied in plant nutrition research [19]. In addition, some studies have used leaf shape focus analysis for plant species identification [20]. Together, these approaches provide useful technical support for precision agriculture. Low-cost and efficient practical technologies are particularly valuable for agricultural applications.

However, studies focusing specifically on tea plant remain limited [21]. Therefore, the objectives of this study were to assess the stability of leaf morphological and color traits among six locally prominent tea cultivars, to analyze inter-cultivar variability in these traits, and to evaluate their potential for accurate cultivar identification. This study provides a basis for leaf trait-based tea cultivar identification and may contribute to the development of automated, vision-based approaches for tea plant recognition, thereby supporting precision agriculture in tea cultivation.

## 2. Materials and Methods

### 2.1. Tea Cultivars

The tea cultivars used in this study were collected from Longtan Village (113.93° E, 32.00° N, approximately 150 m above sea level), Shihe District, Xinyang City, Henan Province, from tea plantations with a cultivation history of 20–60 years. The main cultivars included FuDingDaBai (GS13001-1985), FuDingDaBai variety (corresponding to distinct introduction batches of FuDingDaBai, with tree ages exceeding 30 years), FuYun6, with FuDingDaBai as its parent (GS13033-1987), LongJing43 (GS13007-1987), LaoHan (a local population cultivar of Xinyang), and XiangBoLu (a cultivar from Hunan Province). All tea cultivars were propagated from seeds. All sampled plants were grown under uniform management regimes, with no incidence of pests, diseases, or other plant health issues during the sampling period. Samples were collected randomly from the upper canopy of tea plants to ensure representativeness.

**Figure 1:**
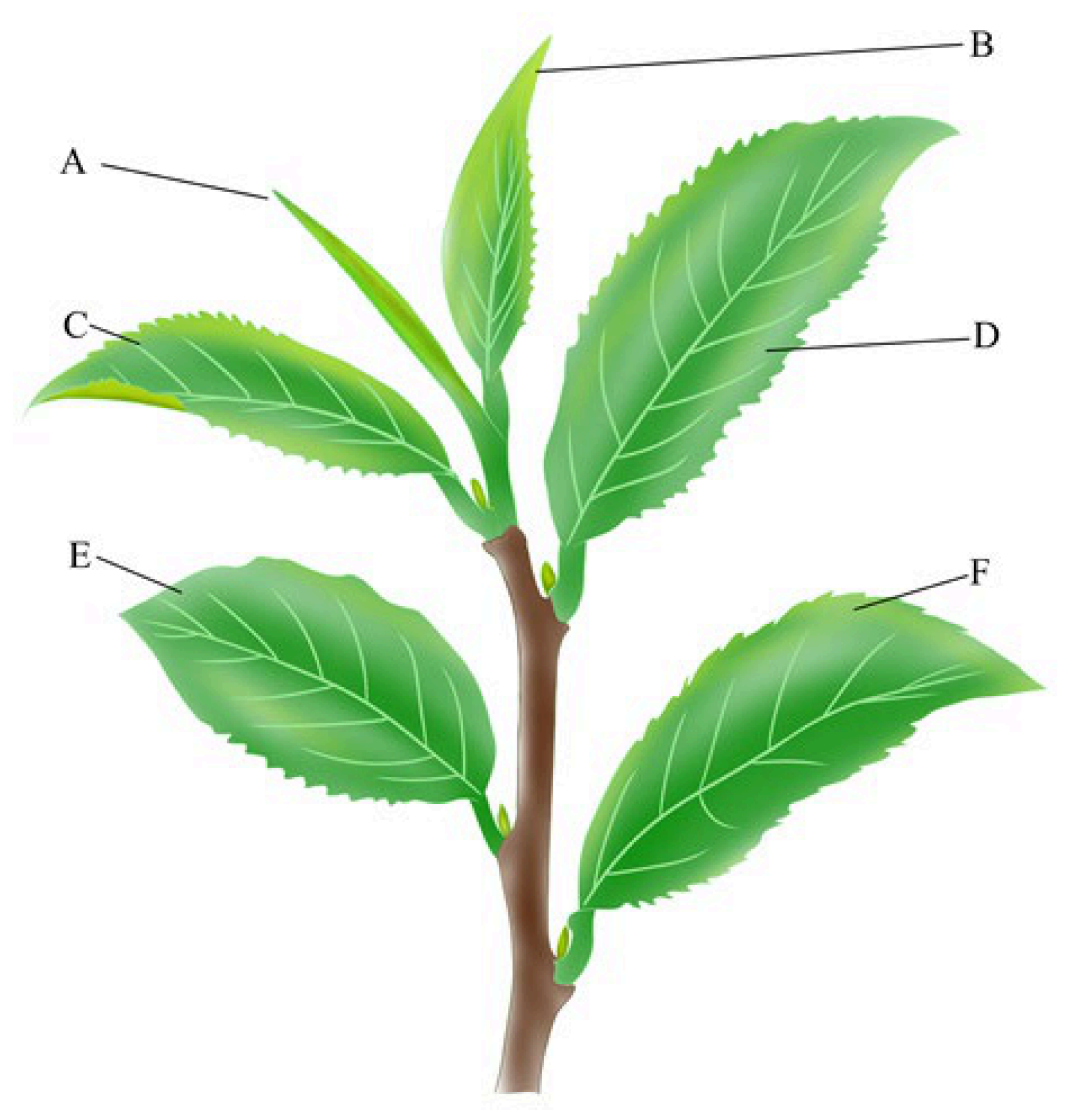
Schematic diagram of leaf position distribution on current-year shoot of tea plants. (A) Bud; (B) first leaf position; (C) second leaf position; (D) third leaf position; (E) fourth leaf position; (F) fifth leaf position. Figure credit: Pengliang Pan.

For sampling, one-year-old shoots bearing 5 fully expanded leaves were selected on 30 November 2025 (the distribution of leaf positions is shown in Figure 1). Within the canopy of each tea bush, five one-year-old shoots were selected randomly within a 3 m range, with no two shoots originating from the same tea tree. For each cultivar, six tea bushes were randomly sampled to ensure consistency in site conditions and management practices across all sampling locations. Thirty shoots were collected as 30 repetitions per cultivar and transported to the laboratory. Subsequently, SPAD value measurements and RGB image acquisitions were performed immediately after the tea leaves excised. Both unprocessed and processed leaves were stored in a refrigerator at 4 °C.

### 2.2. Image Acquisition

**Figure 2:**
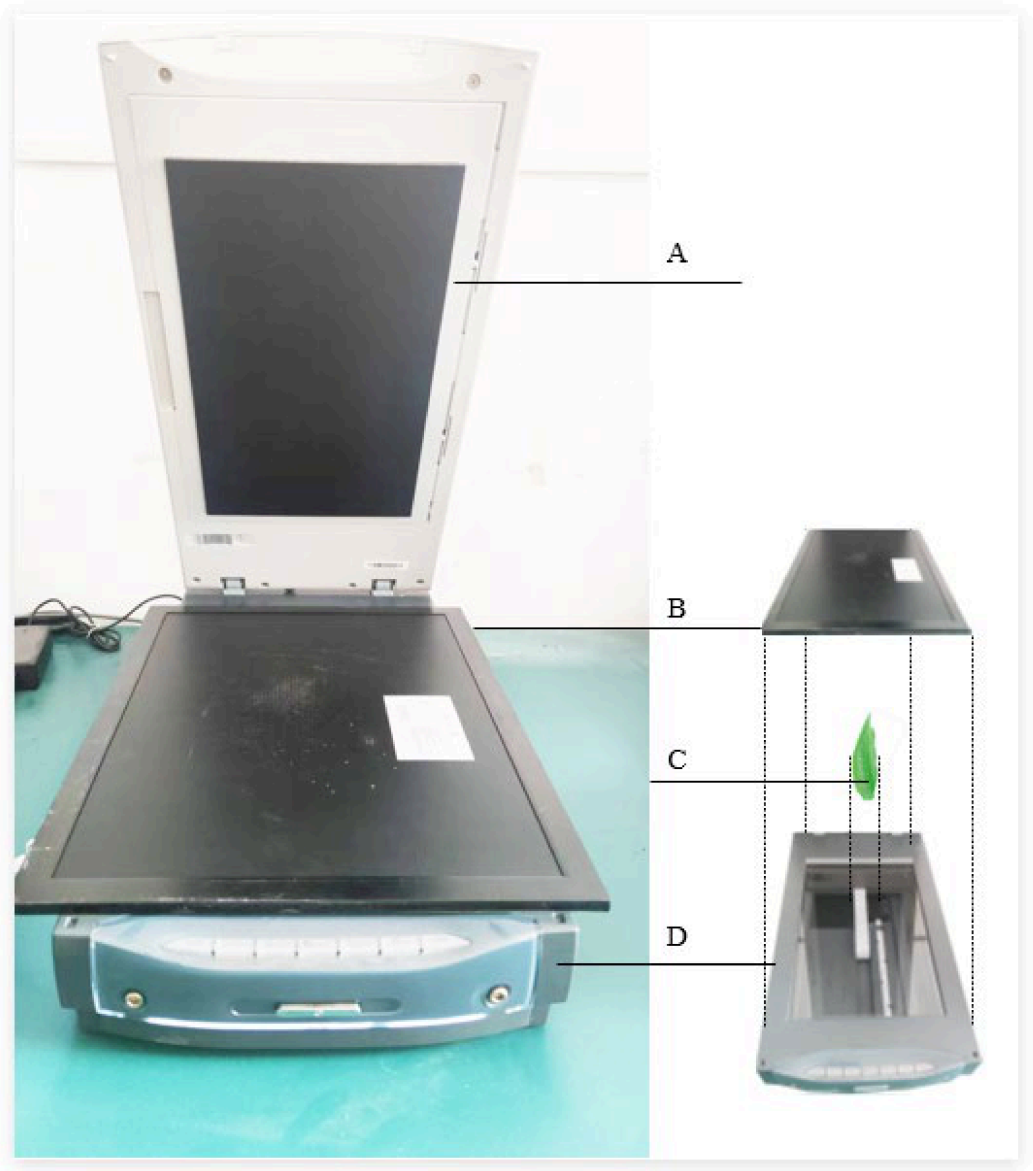
Schematic diagram of the leaf acquisition device and its components. (A) Scanner top cover; (B) light table (light projection direction: downward); (C) tea leaf; (D) scanner working platform. The dashed lines indicate regions where components are in close contact. Figure credit: Pengliang Pan.

In this study, a Microtek MRS-9600TFU2L scanner (Microtek Technology Co., Ltd., Shanghai, China) was employed for tea leaf image acquisition. Tea leaves were excised from shoots along with their petioles, placed on the scanner’s glass platform with the adaxial surface facing downward. The scanning parameters were set as follows: resolution of 2400 dpi, 24-bit color mode for reflective documents, and images saved in JPEG format. The functions including color auto-correction, brightness adjustment, and contrast adjustment were disabled. To minimize leaf shadows, a “Huaxiaomei” copy stand (Model: A3, LED/10W; Shandong Chendouxing Electronic Technology Co., Ltd., Linyi, China) was positioned above the leaves to provide supplementary background illumination (Figure 2).

### 2.3. Feature Extraction

#### 2.3.1. Contour Features

**Figure 3:**
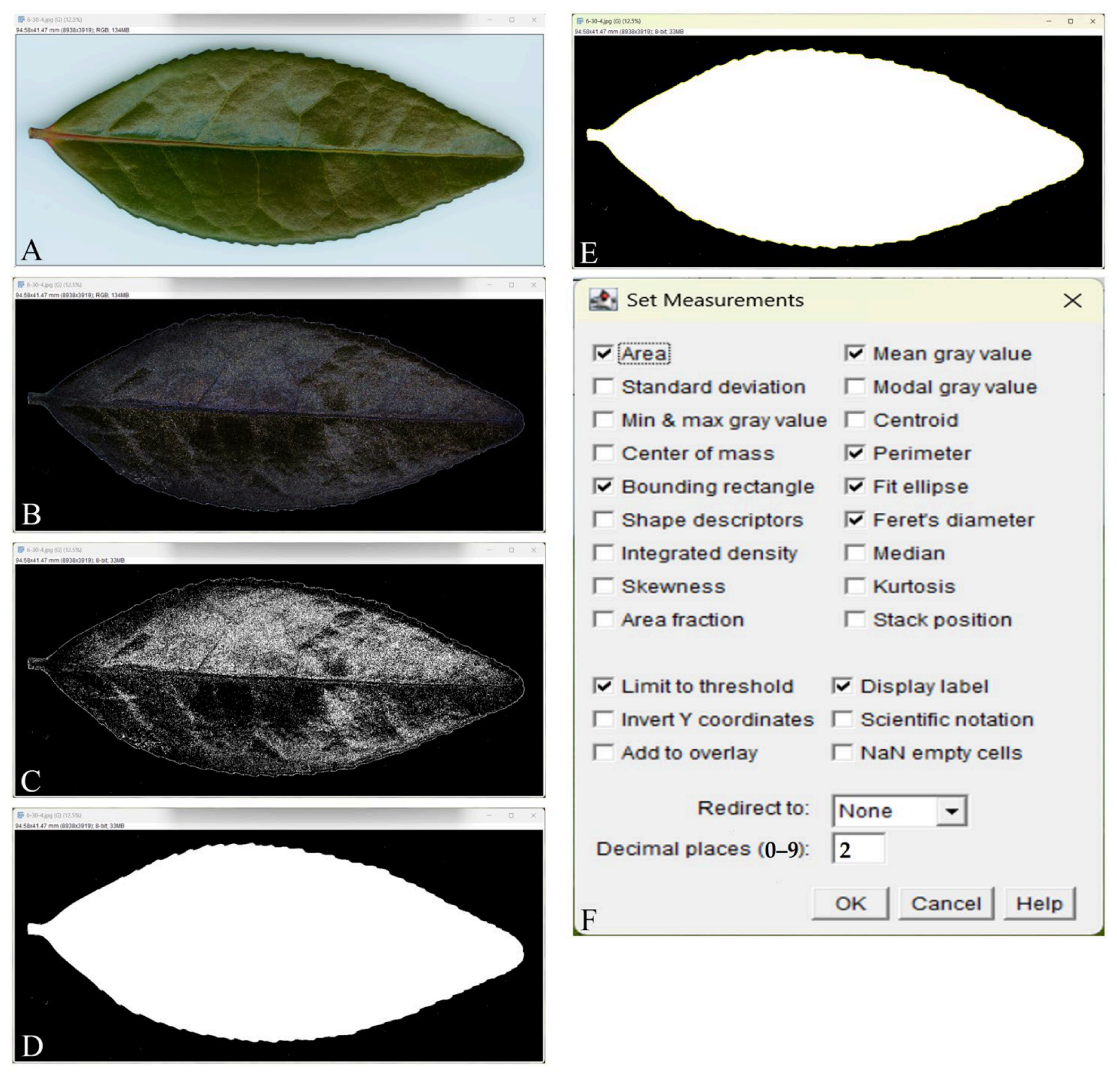
Measurement steps and parameter settings for the morphological characteristics of tea plant leaves in ImageJ v.1.54. (**A**) Loading target image; (**B**) finding edge; (**C**) making binary; (**D**) filling hole; (**E**) getting leaf contour; (**F**) set measurements in ImageJ v. 1.54. Figure credit: ImageJ v. 1.54.

Measurement parameters were configured in the software ImageJ v.1.54 as follows: First, the target image (Figure 3A) was opened, and the actual measurement scale was calibrated via the menu option Analyze → Set Scale. The “Distance in pixels” was set to 94.5, with a corresponding “Known distance” of 1 mm; the “Global” option was enabled to apply this scale uniformly across all measurements, resulting in a calibration of 94.5 pixels/mm. Subsequently, under the Analyze menu, Set Measurements was selected, and the following parameters were checked: Area, Perimeter, Bounding Rectangle, Fit Ellipse, Feret’s Diameter, Limit to Threshold, and Display Label. The number of decimal places was set to 2 (Figure 3F). To ensure the consistency of operational standards, the entire workflow was performed by the same trained experimental technician. Throughout the process, the accuracy of image segmentation was validated by assessing the smoothness of the contour lines.

The image was preprocessed sequentially using Process-Find Edges (Figure 3B), Process-Binary-Make Binary (Figure 3C), and Process-Binary-Fill Holes (Figure 3D) to enhance contour clarity. The leaf contour (Figure 3E) was then delineated using the Wand (tracing) tool. Finally, the Analyze-Measure function was executed to extract contour-related parameters, including leaf area, perimeter, bounding rectangle dimensions (Width for leaf length and Height for leaf width), and the major and minor axis lengths of the fitted ellipse (alternative parameters characterizing leaf length/major axis length and leaf width/minor axis length). When manually delineating contours, the maximum inscribed polygon was constructed by taking the base of serrations on the tea leaf margin as turning points. All operations were executed in strict accordance with a unified protocol by the same trained operator.

#### 2.3.2. RGB Value Extraction

For RGB value acquisition, the Polygon Selection tool in ImageJ v.1.54 was used to manually outline the tea leaf region in each image. The Color Histogram function was then applied to the selected region to extract RGB channel values. All extracted RGB data were recorded in an electronic spreadsheet for subsequent statistical analysis.

#### 2.3.3. Chlorophyll Content Measurement with SPAD Meter

The SPAD-502 Plus chlorophyll meter (Konica Minolta Holdings, Inc., Tokyo, Japan) was employed to measure the relative chlorophyll content (SPAD value) of tea leaves. For each leaf, measurements were taken at three positions: the leaf base, leaf middle, and leaf tip. Three replicate measurements were performed at each position, and the average value was calculated for each leaf. All SPAD data were collated and stored in an electronic spreadsheet for subsequent statistical analysis. Chlorophyll content, quantified via SPAD-502 Plus meter, was assessed immediately upon sample collection to mitigate potential errors arising from excessive chlorophyll degradation.

### 2.4. Statistical Analysis

For tea leaves of different leaf positions or cultivars, descriptive statistics including mean and standard deviation (SD) were calculated for SPAD values and leaf contour feature parameters. One-way analysis of variance (ANOVA) was employed to examine differences between leaf positions and among cultivars, and multivariate general linear models (GLMs) to examine differences in the main effects of tea cultivars and leaf positions. In post hoc comparisons, Tukey’s honestly significant difference (HSD) test was utilized for data with homogeneous variances, whereas the Games–Howell test was applied to data with heterogeneous variances (significance level set at α = 0.05, confidence intervals were set at 95.0%).

Pearson correlation analysis was conducted to quantify the linear relationships between individual RGB channel values and SPAD values. Principal component analysis (PCA) was further employed to integrate multidimensional data—including SPAD values, leaf contour features, and RGB parameters—aiming to characterize and visualize the patterns of phenotypic variation among tea cultivars [22]. Finally, discriminant analysis was employed to assess the performance of the obtained parameters in distinguishing among these tea cultivars. All analyses were performed using SPSS 22.0.

## 3. Results

### 3.1. Morphological Characteristics

#### 3.1.1. Leaf Position-Dependent Variation Patterns Within Cultivars

Levene’s test for homogeneity of variance showed that most parameters across cultivars had homogeneous variances (*df* = 4, 144; *p* > 0.05). Exceptions were observed for FuYun6 for perimeter (*df* = 4, 144; *p* = 0.031), width (*df* = 4, 144; *p* = 0.010), Feret’s diameter (*df* = 4, 144; *p* = 0.010), XiangBoLu (height (*df* = 4, 145; *p* = 0.011)) and minFeret’s diameter (*df* = 4, 145; *p* = 0.009).

**Figure 4:**
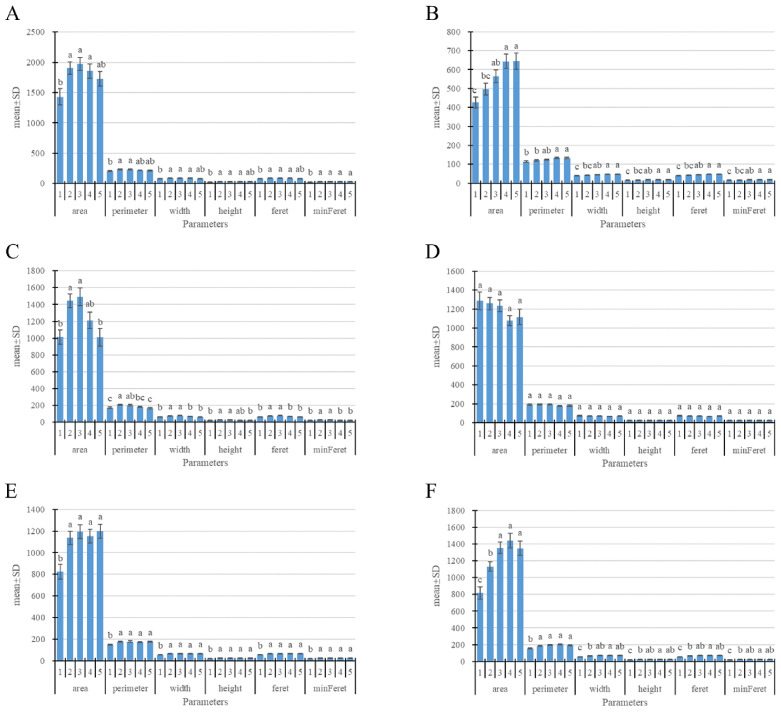
Results of the differences in parameters at different leaf positions (the number in x-axis) for each cultivar (different lowercase letters indicate significant differences among treatments with the same group. α = 0.05). (**A**) FuDingDaBai; (**B**) LaoHan; (**C**) FuDingDaBai variety; (**D**) FuYun6; (**E**) LongJing43; (**F**) XiangBoLu.

One-way ANOVA showed no significant differences in any morphological parameters among leaf positions for FuYun6 (*F* = 0.111–1.672; *df* = 4, 144; *p* > 0.05). In contrast, all other cultivars showed significant leaf position-dependent variation in morphological traits (*p* < 0.05). Specifically, Parameters at the first leaf position were significantly smaller than those at the second leaf position (*p* < 0.05), except for FuYun6 (Figure 4).

For leaf area: LaoHan showed a monotonic increase with advancing leaf position, whereas FuDingDaBai and FuDingDaBai variety showed a unimodal trend with initial increase followed by a decrease. LongJing43 and XiangBoLu had not significant differences in leaf area between the third and fifth leaf positions (*p* > 0.05). Leaf perimeter followed a pattern similar to that of leaf area across cultivars. Parameters representing leaf length such as width and Feret’s diameter and leaf width such as height and minFeret’s diameter showed cultivar-specific variation patterns (Figure 4).

#### 3.1.2. Cultivar-Dependent Variation Patterns

**Figure 5:**
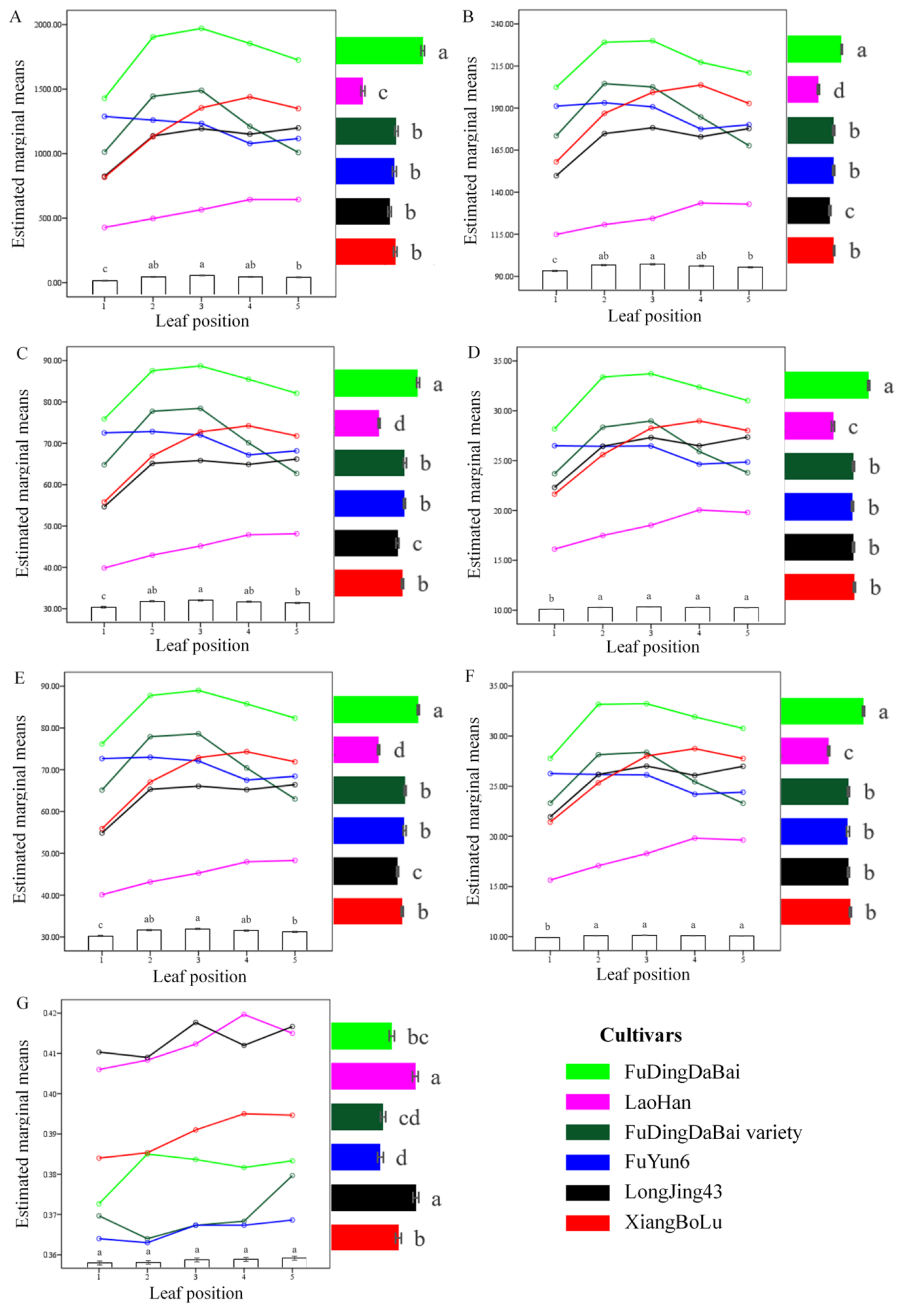
Significant comparisons of differences among various cultivars (colored bar charts) and leaf positions (hollow bar charts), as well as the results of the interaction (colorful lines) between the two fixed factors (cultivars and leaf positions). (**A**) Area; (**B**) perimeter; (**C**) width; (**D**) height; (**E**) Feret’s diameter; (**F**) minFeret’s diameter; (**G**) height/width. Different lowercase letters indicate significant differences among treatments with the same group (α = 0.05).

A two-way general linear model (GLM) was used to evaluate the effects of tea cultivar, leaf position, and their interaction on leaf morphological traits. The model revealed that cultivar had a significant effect on leaf area (η^2^ = 0.402, *F* = 116.913, *df* = 5, *p* < 0.001), perimeter (η^2^ = 0.440, *F* = 136.761, *df* = 5, *p* < 0.001), width (η^2^ = 0.465, *F* = 150.932, *df* = 5, *p* < 0.001), height (η^2^ = 0.384, *F* = 108.367, *df* = 5, *p* < 0.001), Feret’s diameter (η^2^ = 0.466, *F* = 151.537, *df* = 5, *p* < 0.001), and minFeret’s diameter (η^2^ = 0.382, *F* = 107.600, *df* = 5, *p* < 0.001). Leaf positions also had significant effects on these parameters (η^2^ = 0.060–0.076, *F* = 13.851–17.970, *df* = 4, *p* < 0.001); additionally, a significant interaction effect was observed between cultivars and leaf positions (η^2^ = 0.066–0.071, *F* = 3.086–3.309, *df* = 20, *p* < 0.001). For the leaf width-to-length ratio, cultivar had a highly significant effect (η^2^ = 0.153, *F* = 31.292, *df* = 5, *p* < 0.001), whereas leaf position had no significant effect (η^2^ = 0.005, *F* = 1.136, *df* = 4, *p* = 0.338), and the cultivar × leaf position interaction was also not significant (η^2^ = 0.004, *F* = 0.181, *df* = 20, *p* = 1.000) (Figure 5).

Tukey’s honestly significant difference (HSD) tests (α = 0.05) showed that FuDingDaBai had the largest values for leaf area, perimeter, leaf length, and leaf width. In contrast, LaoHan had the smallest values and differed significantly from all other cultivars (*p* < 0.05). No significant differences were observed among FuDingDaBai variety, FuYun6, and XiangBoLu (*p* > 0.05) for any morphological parameters. LongJing43 showed significantly smaller leaf perimeter, leaf width, and fitted ellipse major-axis length than the three cultivars mentioned above (*p* < 0.05), but significantly larger values than LaoHan (*p* < 0.05) (Figure 5).

At the leaf-position level, all morphological parameters (except the width-to-length ratio) at the third leaf position below the bud were significantly larger than those at the first leaf positions (*p* < 0.05) (Figure 5).

### 3.2. Leaf Color Characteristics

#### 3.2.1. SPAD-502 Plus-Based Chlorophyll Relative Content Measurement

Bivariate correlation analysis was performed on SPAD values measured at the leaf base, middle, tip, and their mean. SPAD values measured at all positions were positively correlated with one another (correlation coefficients ranging from 0.961 to 0.979, *p* < 0.01). Specifically, the SPAD value at the leaf base was the highest, that at the leaf tip was the lowest, and that at the leaf middle was closest to the mean of the three positions. Therefore, the mean value was used in all subsequent analyses.

**Figure 6:**
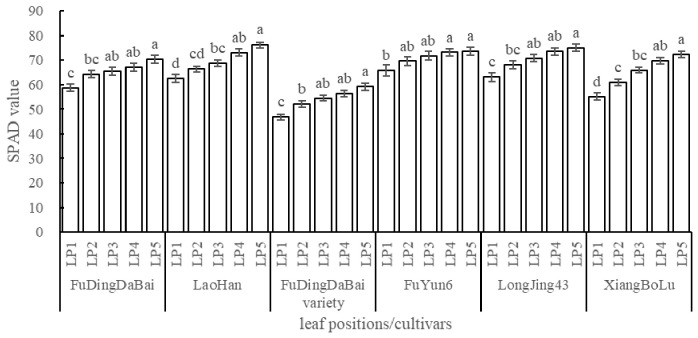
Significant comparisons of SPAD values from different leaf positions (LP1–LP5) for each cultivar (different lowercase letters indicate significant differences among treatments with the same group. α = 0.05).

One-way ANOVA was performed to analyze SPAD values across different leaf positions for each tea cultivar. The SPAD value at the first leaf position of each cultivar was significantly lower than those at all other leaf positions (*p* < 0.05). SPAD values increased progressively with leaf positions. The fourth and fifth leaf positions showed the highest SPAD values, with no significant difference between them (*p* = 0.142) (Figure 6).

Results from the main-effects test of the general linear model (GLM) showed that SPAD values differed significantly among tea cultivars (η^2^ = 0.348, *F* = 92.638, *df* = 5, *p* < 0.001) and among leaf positions (η^2^ = 0.228, *F* = 63.993, *df* = 4, *p* < 0.001), whereas no significant interaction was detected between cultivar and leaf position (η^2^ = 0.020, *F* = 0.895, *df* = 20, *p* = 0.594). Univariate test results showed that the variation among cultivars was significant (*F* = 92.638, *df* = 5, 868, *p* < 0.001), as was the variation among leaf positions (*F* = 63.993, *df* = 4, 868, *p* < 0.001).

Multiple comparisons showed no significant differences between FuDingDaBai and XiangBoLu (*p* = 0.998), LaoHan and FuYun6 (*p* = 0.641), LaoHan and LongJing43 (*p* = 0.968), or FuYun6 and LongJing43 (*p* = 0.977). All other pairwise comparisons between cultivars were highly significant (*p* < 0.001). In addition, highly significant differences were detected across all leaf positions (*p* < 0.01), except for the fourth and fifth leaf positions (*p* = 0.064).

#### 3.2.2. Measurement Based on RGB Images

**Figure 7:**
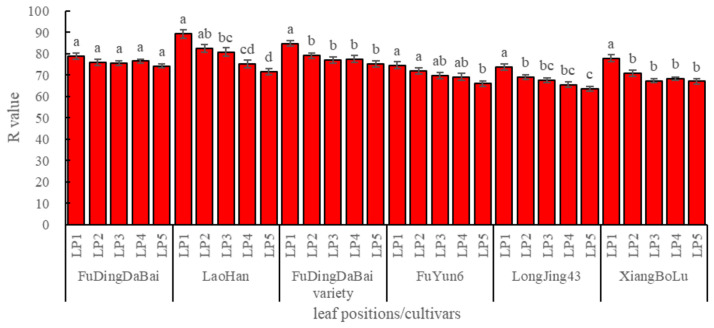
Significant comparisons of R values from different leaf positions (LP1–LP5) for each cultivar (different lowercase letters indicate significant differences among treatments with the same group. α = 0.05).

One-way ANOVA was performed on RGB values for each tea cultivar. For the R value, no significant differences were observed across leaf positions in FuDingDaBai (*F* = 1.836, *df* = 4, 145, *p* = 0.125); in the other cultivars, the R value at the first leaf position was significantly higher than that at the fifth leaf position (*p* < 0.05), and no significant difference was found between the second and third leaf positions (*p* > 0.05) (Figure 7).

**Figure 8:**
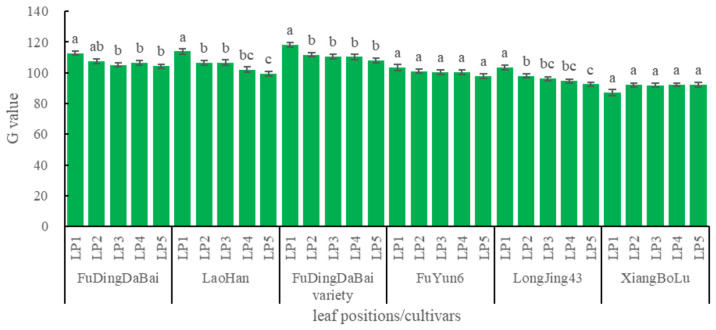
Significant comparisons of G values from different leaf positions (LP1–LP5) for each cultivar (different lowercase letters indicate significant differences among treatments with the same group. α = 0.05).

For the G value, no significant differences were detected across leaf positions in FuYun6 (*F* = 1.566, *df* = 4, 144, *p* = 0.186) and XiangBoLu (*F* = 2.478, *df* = 4, 145, *p* = 0.047). The *p*-value of the homogeneity of variance test was 0.022; thus, Games–Howell multiple comparisons were conducted, whereas for the remaining cultivars, the G value at the first leaf position was significantly higher than those at other leaf positions (*p* < 0.05), with the exception of FuDingDaBai, where no significant difference was observed (Figure 8).

**Figure 9:**
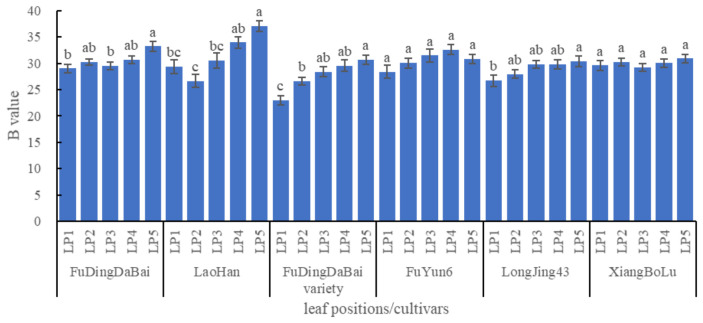
Significant comparisons of B values from different leaf positions (LP1–LP5) for each cultivar (different lowercase letters indicate significant differences among treatments with the same group. α = 0.05).

For the B value, no significant differences were detected across leaf positions in XiangBoLu (*F* = 0.617, *df* = 4, 145, *p* = 0.651) and FuYun6 (*F* = 2.176, *df* = 4, 144, *p* = 0.075); in the other cultivars, the B value at the fifth leaf position was significantly higher than that at the first leaf position (*p* < 0.05) (Figure 9).

In the GLM, RGB values exhibited statistically significant effects of cultivar, leaf position, and their two-way interaction (all *p* < 0.01). Effect sizes (partial η^2^) and corresponding *F*-statistics for the R, G, and B channels were as follows:

Cultivar: partial η^2^ = 0.261, 0.422, 0.057; *F*(5) = 61.357, 126.660, 10.435;

Leaf position: partial η^2^ = 0.165, 0.088, 0.090; *F*(4) = 43.058, 20.885, 21.510;

Cultivar × leaf position interaction: partial η^2^ = 0.042, 0.072, 0.064; *F*(20) = 1.928, 3.359, 2.972.

### 3.3. The Relationship Between SPAD Values and Color Feature Parameters

To examine the relationship between measured SPAD values and color features, bivariate correlation analysis was performed. SPAD values were significantly negatively correlated with the R value (the Pearson correlation coefficient was −0.510, *n* = 898, *p* < 0.001) and the G value (the Pearson correlation coefficient = −0.529, *n* = 898, *p* < 0.001), but significantly positively correlated with the B value (The Pearson correlation coefficient = 0.475, *n* = 898, *p* < 0.001).

### 3.4. Principal Component Analysis

**Figure 10:**
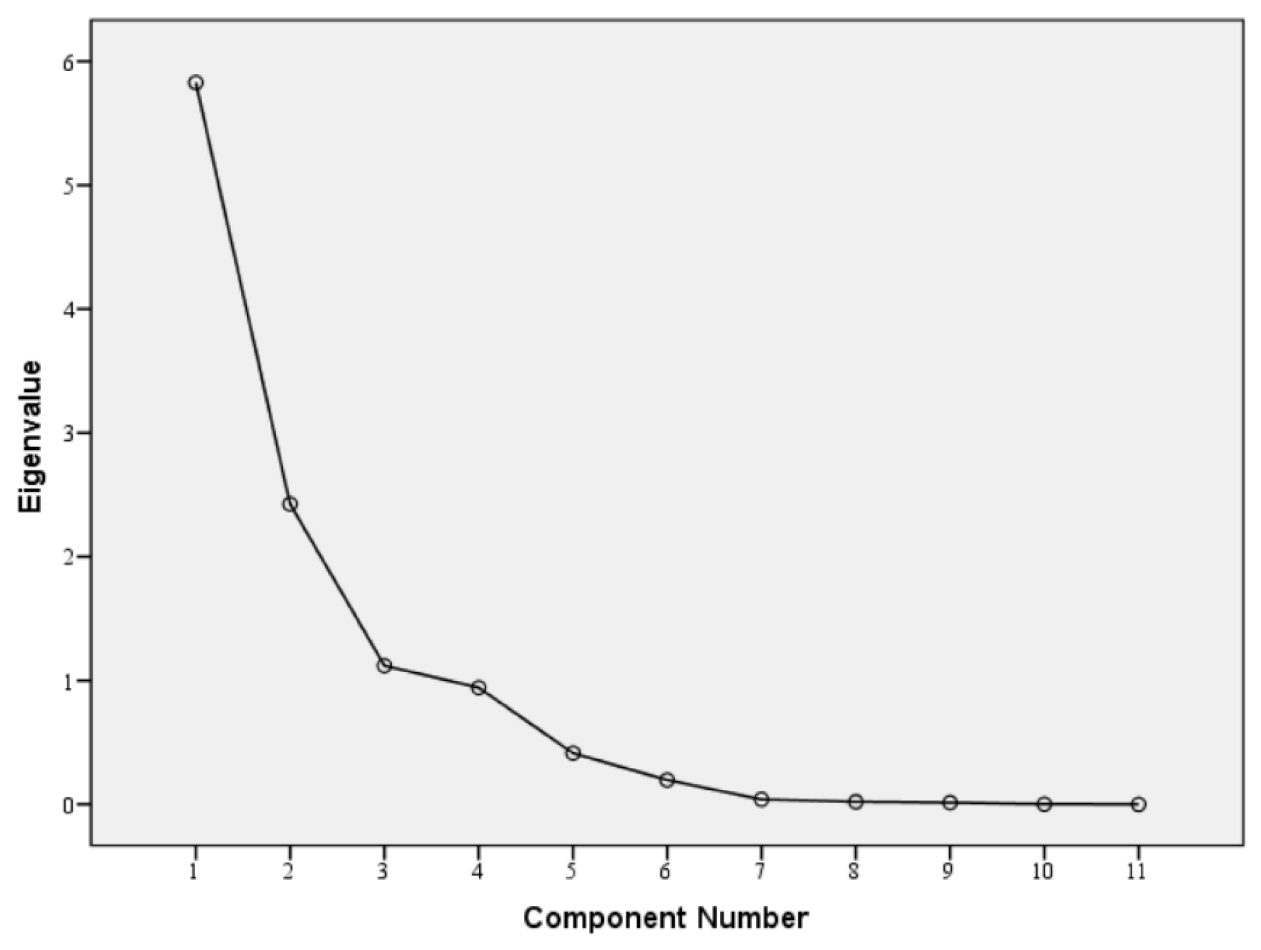
The scree plot generated by principal component analysis.

**Figure 11:**
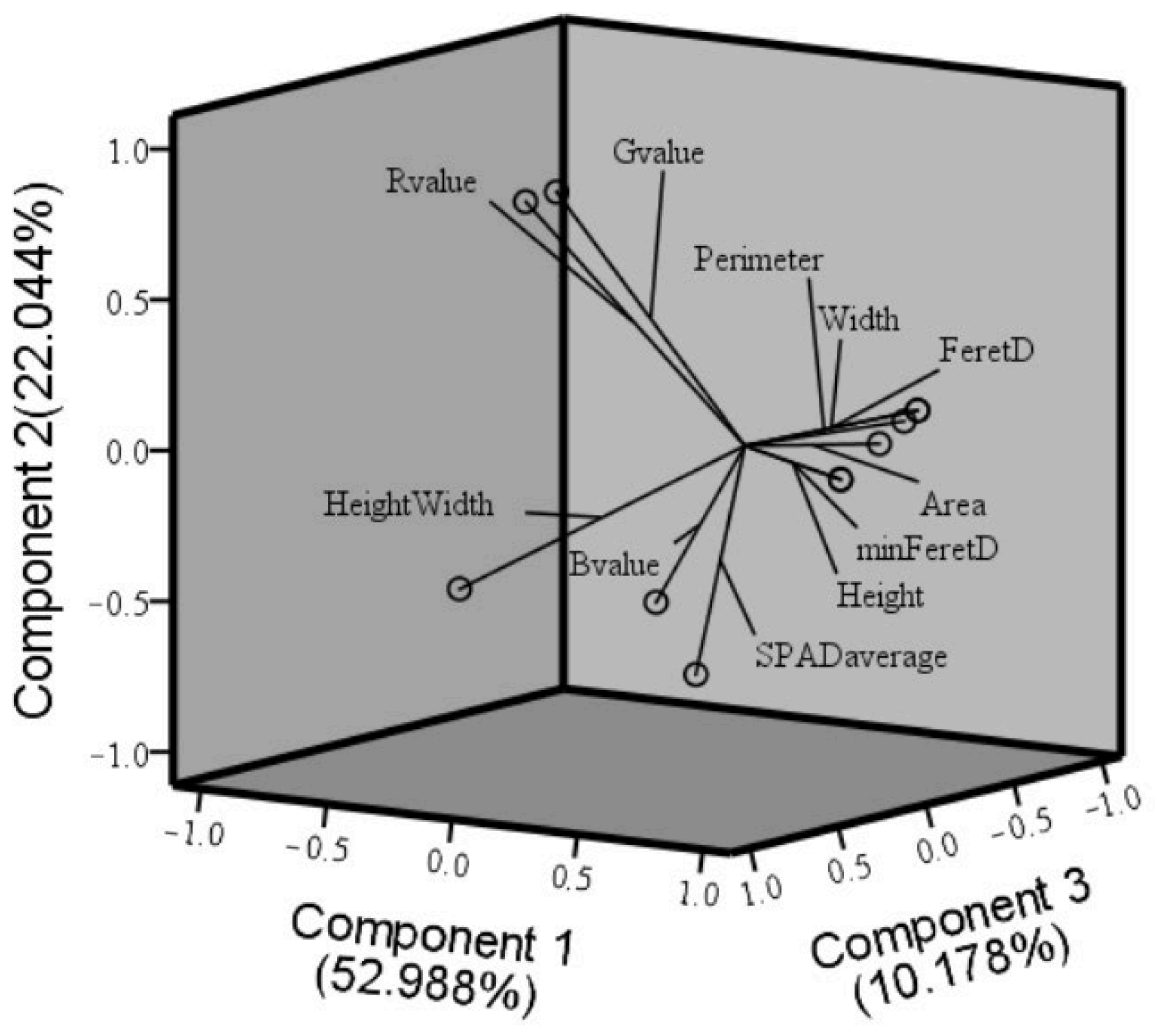
Component plot of factors from principal component analysis.

Based on the above results, principal component analysis (PCA) was performed on the measured SPAD values, RGB values, and leaf morphological parameters. The Kaiser–Meyer–Olkin (KMO) measure of sampling adequacy was 0.789, and Bartlett’s test of sphericity yielded *p* < 0.001, confirming that the data were suitable for PCA. The first three principal components (PCs) explained 52.988%, 22.044%, and 10.178% of the variance, respectively, with a cumulative variance contribution of 85.210% (Figure 10). PC1 represented leaf morphological traits of tea leaves, excluding secondary variables; PC2 represented color-related features including SPAD values and RGB values, and PC3 corresponded to the secondary variable, namely the leaf length-to-width ratio (Table 1, Figure 11).

### 3.5. Discriminant Analysis for Tea Cultivar Classification

Discriminant analysis was performed on all samples, and the first five canonical discriminant functions were used in the analysis. The contribution rates of the first three discriminant functions were 45.6%, 31.2%, and 11.4%, respectively, with a total cumulative contribution rate of 88.2%. Cross-validation via the classification functions yielded an overall discriminant accuracy of 64.6%. The cross-discriminant accuracy of LaoHan reached 83.3%, whereas that of FuDingDaBai variety was 68.7% and XiangBoLu was 69.3%. The accuracies of FuDingDaBai, FuYun6, and LongJing43 were lower, at only 56.0%, 57.4%, and 52.7%, respectively. Among the misclassified samples, the inter-cultivar misclassification rates were relatively high between FuDingDaBai and FuDingDaBai variety, and between LongJing43 and FuYun6 (Table 2).

## 4. Discussion

The results of this study demonstrated substantial intraspecific variation in leaf morphology among leaf positions in most tea cultivars, with distinct position-dependent patterns among cultivars. Both cultivar and leaf position had statistically significant effects on leaf morphological parameters, and their interaction was also significant. In contrast, the leaf width-to-length ratio was significantly affected by cultivar but not by leaf position, and no significant interaction was detected for this trait. Among the cultivars tested, FuDingDaBai and its variant share identical genetic backgrounds, whereas FuYun6 (developed through single-plant selection from the natural hybrid progeny of FuDingDaBai and YunNanDaYe) is partially related to these two cultivars. While molecular identification was not performed in this study, tea farmers have confirmed that tea plants with distinct denominations showed notable differences in their field traits, leading them to recognize these as distinct varieties. Accordingly, we proposed the observed differences in morphological variation were consistent with their degree of genetic divergence. Discriminant analysis further showed higher misclassification rates among cultivars with homologous genetic backgrounds, consistent with previous findings [23].

For leaf color, the mean SPAD value served as a reliable proxy for leaf greenness, with values at the first leaf position significantly lower than those at subsequent positions and increasing progressively with leaf position. Both cultivar and leaf position significantly affected SPAD values, whereas no significant interaction was detected. This result suggests that, as in other plant species, tea leaves show positional/age-related variation in chlorophyll content [24,25]. Notably, SPAD values were significantly negatively correlated with RGB red (R) and green (G) components, but significantly positively correlated with the blue (B) component. This pattern differs from that reported for potato leaves [26], suggesting that the relationship between digital RGB values and SPAD-derived chlorophyll content may be species specific.

While the overall cultivar classification accuracy (64.6%) was modest—with the highest accuracy (83.3%) observed for LaoHan—this study confirms the utility of leaf morphological and color traits in tea cultivar discrimination. The main factor limiting classification accuracy was the high genetic homology among half of the tested cultivars.

With the advancement of smart agriculture, automated, high-throughput detection of plant growth status and cultivar identity has emerged as a key research focus, with leaf traits serving as a critical target for such analyses [27,28]. Previous studies have confirmed that SPAD values are a reliable indicator of chlorophyll concentration across plant species [29]. However, SPAD readings are sensitive to genotype, water status, and nitrogen levels [13,30], as well as to leaf age [25], position [24], and even bilateral asymmetry [31]. Although leaf position on new shoots was considered in this study, potential bilateral asymmetry along the leaf midrib was not investigated in tea plants.

In the context of SPAD prediction via image processing, some studies advocate for increasing parameter complexity to enhance RGB model accuracy [32], while others highlight the utility of color indices [33]. In addition, research integrating leaf contour features have shown that closely related tea cultivars derived from the same parental lineage are difficult to distinguish using contour traits alone [23]. Accordingly, this study integrated leaf contour features, RGB values, and SPAD data, but did not examine color index-based approaches. Methodologically, the use of a chlorophyll meter for SPAD measurements, a desktop scanner for image acquisition, and ImageJ v.1.54 for RGB extraction is generally considered reliable [34]. For color correction, laboratory settings with controlled conditions may not require calibration; however, field-based measurements necessitate correction—despite some studies challenging the need for standard calibration boards [35]. As costs continue to decline, deep learning [7] and hyperspectral imaging [6] are likely to become increasingly important in smart agriculture. Nevertheless, complementary studies on alternative traits and technologies for cultivar discrimination remain necessary.

Although all sampling sites in this study were managed under the same protocols, the sampled tea cultivars differed in age and were grown in a randomly mixed multi-cultivar system. This study therefore focused only on the performance of individual cultivars under local cultivation practices within mixed plantations. No samples were collected from monoculture plots or seedlings [36], and thus the possibility that long-term inter-cultivar interactions influenced leaf morphological and other phenotypic traits cannot be excluded. Furthermore, the sampled plants were in a healthy state under field conditions, and their leaf morphology and coloration were therefore considered representative of the respective cultivars. However, phenotypic variation under abiotic stress, such as drought, was not assessed [37].

In summary, this study has certain limitations in the sampling of tea cultivars. Specifically, molecular identification was not performed on the cultivars, and instead, the classification relied on grower’s empirical references and the filed performance of the cultivars. Additionally, there were differences in the cultivation ages among the various tea cultivars, and the potential for inter-cultivar interference within the mixed-cultivation system was not evaluated. However, the causes of these limitations and their impacts on the study have been addressed through the experimental methodology and discussion. It is worth noting that the comprehensive use of morphological traits, color traits, and chlorophyll content to assess inter-cultivar differences in a mixed-cultivation system is a valuable undertaking. The application of visual technology in this study to characterize variation in morphological and color traits among tea cultivars not only facilitates differentiation among existing tea varieties and provides baseline data for precision agriculture, but also offers potential insights for testing hypotheses concerning geometric morphological changes during plant variety evolution [38].

## 5. Conclusions

An exploratory analysis was conducted on leaf morphological traits, RGB values, and SPAD values of six tea cultivars. The results revealed significant differences in leaf morphological traits both among cultivars and across leaf positions, with a significant interaction effect between cultivar and leaf position. Although cultivar exerted a significant effect on the leaf width-to-length ratio, this trait remained relatively stable across different leaf positions among these cultivars. SPAD values exhibited a gradual upward trend from the first leaf position to subsequent leaf positions, and were significantly negatively correlated with red (R) and green (G) values, but significantly positively correlated with blue (B) values.

In conclusion, based on the six tea plant cultivars examined in this study, leaf morphological traits contribute to the understanding of varietal differentiation, and the integration of multidimensional leaf characteristics shows potential for the discrimination of tea cultivars. Future studies that incorporate additional external traits such as leaf surface texture, vein distribution patterns, and more nuanced features [39] may help enhance the accuracy of identifying closely related tea cultivars.

## Figures and Tables

**Table 1:** Component matrix from principal component analysis.

Parameters	Components		
	1	2	3
Area	0.981		
Perimeter	0.974	0.137	
Width (W)	0.972	0.161	−0.152
Feret’s diameter	0.972	0.161	−0.151
Height (H)	0.960		0.274
minFeret’s diameter	0.959		0.267
G value	−0.225	0.815	0.194
SPAD	0.107	−0.798	−0.124
R value	−0.334	0.775	0.214
B value		−0.542	
H/W	−0.114	−0.388	0.904

**Table 2:** Discriminant analysis results for all tea plant cultivars (leave-one-out classification).

Validation Type *	Cultivar	Predicted Group Membership						Total
		FuDingDaBai	LaoHan	FuDingDaBai Variety	FuYun6	LongJing43	XiangBoLu	
Original validation (%)	FuDingDaBai	57.3	0.7	24.0	13.3	4.7	0.0	100.0
	LaoHan	0.0	86.0	1.3	7.3	5.3	0.0	100.0
	FuDingDaBai variety	13.3	3.3	69.3	8.0	4.0	2.0	100.0
	FuYun6	8.1	5.4	15.5	57.4	10.8	2.7	100.0
	LongJing43	4.0	8.7	7.3	20.7	55.3	4.0	100.0
	XiangBoLu	0.7	2.0	1.3	11.3	14.7	70.0	100.0
Cross-validation (%)	FuDingDaBai	56.0	0.7	24.0	14.0	5.3	0.0	100.0
	LaoHan	0.0	83.3	1.3	8.7	6.0	0.7	100.0
	FuDingDaBai variety	13.3	3.3	68.7	8.7	4.0	2.0	100.0
	FuYun6	8.1	5.4	15.4	57.4	10.8	2.7	100.0
	LongJing43	4.0	8.7	8.0	22.7	52.7	4.0	100.0
	XiangBoLu	0.7	2.0	2.0	11.3	14.7	69.3	100.0

* 65.9% of original grouped cases are correctly classified and 64.6% of cross-validated grouped cases are correctly classified.

## Data Availability

The original contributions presented in this study are included in the Supplementary Materials.

## Supplementary Materials

The following supporting information can be downloaded at https://www.mdpi.com/article/10.3390/biology15151283/s1, Table S1: Original data of leaf morphological traits, RGB values, and SPAD values from six tea cultivars in this study.

## Abbreviations

The following abbreviations are used in this manuscript:

SPAD	Soil–Plant Analysis Development
RGB	Red, Green, Blue
SD	Standard Deviation
ANOVA	Analysis of Variance
LSD	Least Significant Difference
PCA	Principal Component Analysis
GLM	General Linear Model
LP	Leaf Position
KMO	Kaiser–Meyer–Olkin
PC	Principal Component
